# Estrogen Receptors and Melanoma: A Review

**DOI:** 10.3390/cells8111463

**Published:** 2019-11-19

**Authors:** Emi Dika, Annalisa Patrizi, Martina Lambertini, Nicholas Manuelpillai, Michelangelo Fiorentino, Annalisa Altimari, Manuela Ferracin, Mattia Lauriola, Enrica Fabbri, Elena Campione, Giulia Veronesi, Federica Scarfì

**Affiliations:** 1 Dermatology Section, Department of Experimental, Diagnostic and Specialty Medicine, DIMES, University of Bologna, 40138 Bologna, Italy; annalisa.patrizi@unibo.it (A.P.); martinalambertini@gmail.com (M.L.); nick@mpillai.com (N.M.); giulia.veronesi.md@gmail.com (G.V.); scarfif@gmail.com (F.S.); 2 Pathology Unit, Department of Experimental, Diagnostic and Specialty Medicine, DIMES, University of Bologna, 40138 Bologna, Italy; michelangelo.fiorentino@unibo.it (M.F.); manuela.ferracin@unibo.it (M.F.); enrica.fabbri@unife.it (E.F.); 3 Laboratory of Oncologic Molecular Pathology, S.Orsola-Malpighi Hospital, 40138 Bologna, Italy; annalisa.altimari@aosp.bo.it; 4 Histology, Embryology and Applied Biology Unit Department of Experimental, Diagnostic and Specialty Medicine—DIMES University of Bologna, 40138 Bologna, Italy; mattia.lauriola2@unibo.it; 5 Division of Dermatology, Department of Systems Medicine, University of Rome Tor Vergata, 00133 Rome, Italy; campioneelena@hotmail.com

**Keywords:** melanoma, woman, estrogens

## Abstract

In the last three decades cutaneous melanoma has been widely investigated as a steroid hormone-sensitive cancer. Following this hypothesis, many epidemiological studies have investigated the relationship between estrogens and melanoma. No evidence to date has supported this association due to the great complexity of genetic, external and environmental factors underlying the development of this cancer. Molecular mechanisms through which estrogen and their receptor exert a role in melanoma genesis are still under investigation with new studies increasingly focusing on the discovery of new molecular targets for therapeutic treatments.

## 1. Introduction

Melanoma incidence continues to increase globally, giving rise to many questions about its exact pathogenesis [1]. Intriguingly, to date, most of the epidemiological data has noted a significant gender divergence in melanoma incidence as well as in other cancers. Indeed, in the last decades, melanoma incidence has been increasing more rapidly among males compared to females in all age categories, except for young women (≤39 years) who appear to be at higher risk. In line with these observations, slightly higher rates of melanoma have been reported for young women (20–45 years) which subsequently decreases after 45 years of age. On the other hand, in males, melanoma incidence progressively increases after 50 years of age [2,3,4,5].

The gender difference in melanoma survival appears to be limited to the early stages of the disease, since no difference in survival has been detected in advanced metastatic melanoma by the Surveillance, Epidemiology and End Results (SEER) database. This analysis was based on 106,511 cases of melanoma collected over 20 years in the United States, but not confined to pre- or perimenopausal age groups [5]. Confirming that for melanoma, women have a significant survival advantage over men [6]. Nowadays, this data is particularly relevant as women continue to delay childbearing into their 30′s and 40′s, increasing the likelihood of melanoma diagnosis during pregnancy [7]. Indeed, a meta-analysis of epidemiological studies highlighted that the risk of cutaneous melanoma is positively associated with the age of first pregnancy and inversely associated with parity [8]. Recent studies have suggested an important area of research to be female endogenous hormone exposure from menarche to menopause and its correlation with ultraviolet radiation [9].

The reasons why women seem to have a better prognosis are not completely understood. A large body of epidemiological studies have verified that gender is an independent prognostic factor after adjusting for other known melanoma risk factors such as: Breslow thickness, ulceration, histologic subtype, location, sentinel lymph node positivity and age [10,11,12]. Gender disparity in melanoma outcomes are consistently observed, leading to the suggestion that gender status should be incorporated in staging algorithms [13]. The gender disparity may be partially due to the differences in behavior and biology between the two genders. For example, women tend to have more ultraviolet protection and more frequent medical visits compared to men, who are more likely to have thicker, ulcerated tumors, as a result of diagnostic delays [14,15]. However, these factors don’t fully explain why gender is still a key prognostic factor after considering the gap between different lifestyle and healthcare delivery [16,17]. Regarding biology, many studies have pointed to differences in pharmacokinetics, immune response/inflammation and hormones between genders [18,19]. As is well known, there are gender differences in all the main pharmacokinetic parameters such as the absorption, distribution, metabolism, and elimination of drugs, all of which could impact the outcome of the disease [20].

Although melanoma is classically considered a non-hormone-related cancer, increasing evidence support a direct correlation between sex hormones (estrogens, in particular) and melanoma. Estrogen was found to play a role in the female survival advantage that seems to be abrogated in the postmenopausal period when estrogen levels decrease [20,21]. Additional evidence report that gender has an impact only in local tumor invasion [22], and that the gender effect is limited to lymphatic or hematogenous metastasis [23,24]. There are limited studies that consider metastatic patients, although, the results remain controversial [5,6,25,26]. The role of the immune system is crucial, particularly in the era of anticancer immunotherapy. There is evidence of a clear immunological difference between genders. Women have a better response in terms of effectiveness in both cellular and humoral immunity and exhibit a complex plasticity during the pregnancy, enabling them to “tolerate” a genetically different organism inside their bodies [19].

## 2. The Source of Data

The present review was conducted and reported using validated search strategies from the following databases:

- PUBMED
- Ovid MEDLINE
- ISI Web of Science

The keywords and/or MESH terms used were: estrogen/oestrogen, estrogen/oestrogen receptors, and melanoma. Additional studies were found through cross-referencing of reference lists of the retrieved articles and previous reviews on the topic. Our search was not limited to human studies as it also considered molecular studies.

## 3. Estrogens and Melanoma: Epidemiological Studies

The distinct gender differences in relation to survival, local tumor invasion and more controversially, metastatic spread, warrant further investigation of the assumption that melanoma is a non-hormone related cancer [22,23,24,25,26]. Furthermore, the reduction of the survival advantage in postmenopausal women with melanoma provides an additional indication of the important immunological role of estrogen in relation to melanoma genesis and treatment [21,22].

More recent clinical, epidemiological, and laboratory studies have shed some light on the relationship among hormones, nevi, and melanoma in pregnancy. Similarities between the pathologic mechanisms of cancer and the physiologic process of placentation (e.g., proliferation, invasion, and local/systemic tolerance) have been researched for many years. Sex hormones such as human chorionic gonadotropin, estrogens, progesterone, and others have been shown to play contributory roles in the growth of sex hormone sensitive cancers such as breast, prostrate, endometrial and ovarian tumors [27]. Despite this, the involvement of sex hormones as putative mediators of the immunologic escape of cancer are still not known.

The sex hormones may be involved in a mechanism responsible for the immunotolerant environment during pregnancy, which blocks maternal T-cell responses and prevents fetal rejection. Indeed, progesterone and estrogen are important regulators of the immune system and angiogenesis in several types of cancers and in pregnancy [27,28]. Additionally, androgen receptors have been detected in melanoma cells, raising the hypothesis of its role in melanoma among men [29]. Questions regarding the relationship between the role of sex steroid hormones and melanoma have prompted studies examining epidemiological data about the impact of estrogen use for therapeutic purposes in patients with melanoma. A US study of 167,503 women, found a strong association between ambient UV exposure and melanoma in women who experienced menarche at an early age and menopause at a late age [9], further suggesting an important hormone related role in melanoma pathogenesis.

The possible link between exogenous sex steroid consumption and melanoma development has also been extensively investigated. Exogenous female hormones, either as oral contraceptives or as hormonal replacement among patients with a personal history of melanoma, have been investigated in order to find an association with the risk of developing melanoma. There is no convincing evidence that both the hormone replacement therapy and the oral contraceptive pill, effect the risk of developing melanoma. Moreover, hormone replacement therapy and oral contraceptives are not contraindicated in women who have had melanoma [30,31,32,33,34,35,36,37,38,39,40,41,42,43,44,45]. Recently a cohort study of 684,696 Norwegian women, found an increased risk of melanoma among menopausal women using estrogen-only hormone therapy (HT) (tablet and vaginal forms). Whereas, combined estrogen–progestin HT was not associated with an increased risk. This finding seems to suggest exogenous estrogen as a risk factor for melanoma. More studies are required to confirm these findings [46].

Several studies with conflicting results have focused on fertility drugs as drivers of melanoma. During in vitro fertilization (IVF) women are exposed to levels of estrogen and progesterone that can be 10 times greater than those present physiologically [47]. Previous studies have demonstrated an increased incidence of melanoma in women undergoing controlled ovarian stimulation (COS) [48,49] especially in relation to clomiphene citrate, a drug used in COS. Conversely, a number of studies have also found no association between malignant melanoma risk and the use of fertility drugs [50,51,52,53,54,55].

## 4. Estrogens and Melanoma: Molecular Studies

**Figure 1:**
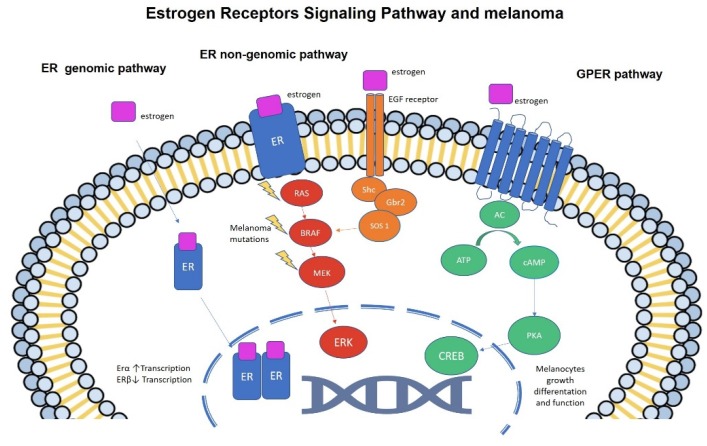
The main estrogen receptors signaling pathways: in the genomic pathway, classic estrogen receptors (ER α and β) act as transcription factors upon homo- or heterodimers. ER α promotes DNA transcription, while ERβ inhibits it. In the non-genomic pathway ERs act as membrane receptors and they lead to increased activity of the RAS/BRAF/MEK axis. Figure 1 demonstrates cross-talk with EGFR receptor pathway. The G protein-coupled estrogen receptor (GPER) acts via intracellular cAMP-protein kinase (PK) and cAMP-response element-binding protein (CREB) phosphorylation.

Steroid hormones such as estrogen act through their cognate receptors: Estrogen Receptor alfa (ERα) and Estrogen Receptor beta (ERβ). ERs belong to the nuclear receptor superfamily, which act as transcription factors upon homo- or heterodimers. Estrogen binding to the nuclear receptors are responsible for a nuclear translocation, with consequent activation of genomic pathways and transcription of multiple target genes. More rapid effects of steroid hormones are elicited by a “non-genomic pathway” activation. In this latter case, membrane-bound estrogen receptors are responsible for change in the cytosolic signaling, leading to increased activity of the RAS/BRAF/MEK axis [56] (Figure 1).

Notably, targeting the MAPK pathway by the BRAF inhibitor, vemurafenib, or the MEK inhibitor, dabrafenib, have been approved by the FDA for V600E mutant BRAF-harboring melanomas. Mutant BRAF is an important driver in melanoma development, occurring in about 50% of cutaneous melanomas. Thus, the cross-talk of steroid hormones with the route responsible for the melanoma transformation should be thoroughly addressed in order to determine the exact involvement of steroid hormones in melanoma development. The physiological relevance of the cross-talk of the steroid driven non-genomic pathway and MAPK activation was clearly demonstrated in an ERα KO mice model. Where the uterus displayed a growth response upon MAPK activation by epithelial growth factor. This action was not present with the absence of ERα, suggesting a key interaction between these two pathways. This is not surprising given a broad body of literature has suggested a coupling interaction of steroid hormones and growth factor receptor, with a fine-tuning of the MAPK/ERK axis, strongly impacting on cellular output in physiological or pathological conditions [57,58].

The estrogen receptors are encoded by two different genes that are located on chromosomes 6 and 14. Synthetic or natural ligands bind to ERα or ERβ with different affinities according to their chemical structure [56,57]. Increasing evidence supports a relationship between the perturbation of estrogen signaling and cancer initiation, promotion, and progression. Overall, it is now well accepted that ERα plays a role in tumorigenesis by stimulating cell proliferation, while ERβ seem to have a significant antitumor activity [57]

ERα is the main estrogen receptor in the human epidermis and its expression is decreased in benign or dysplastic nevi, in metastatic or primary melanoma, as well as in pregnancy-associated melanoma. Thus, the overall abundance of the receptor does not appear to be associated with the pathophysiology of melanoma precursor lesions or melanomas [59]. However, a recent immunohistochemical study reported a cytosolic localization of ERα in melanoma of women who underwent more than one cycle of in vitro fertilization [60]. In all individuals the probability of inherited susceptibility to melanoma could be partially due to different genomic ERα single nucleotide polymorphisms. These polymorphisims have been found to impact the course of the disease as well as the response to chemotherapy [61].

On the other hand, ERβ has been reported to be the predominant ER subtype in all melanocytic lesions such as moles, dysplastic nevi and melanoma [62]. Furthermore, ERβ expression is diminished after in vitro cellular exposure to UV and is associated with thinner lesions with a prominent epidermal component, such as severely dysplastic nevi or melanoma in situ. It has been suggested that ERβ could represent a marker for metastatic potential and prognosis in malignant melanoma [63]. ERβ receptor appears inversely associated with an increased Breslow depth [64] and displays a protective role in the metastatic process. Similar data has been reported in breast, prostate and ovarian tumors and colon cancers [65,66,67,68,69,70,71,72]. Furthermore, ERβ has been found to be more highly expressed in melanomas of pregnant women than in men, and a trend indicating a higher expression among women in comparison to men has been shown. This result was obtained by evaluating hormone receptor expression in the melanomas of stage and age-matched patients of pregnant women, non-pregnant women, and men [73].

An equally important type of estrogen receptor is the G protein-coupled estrogen receptor (GPER). GPER belongs to G protein-coupled receptor family of cellular membrane molecules that are found to be involved in development and progression of different cancer types [74]. GPER has specific agonists, G1 and 17β-estradiol. It is responsible for many cellular functions. In skin, it regulates melanin production and is expressed in melanoma cells [75]. It promotes melanogenesis via an intracellular pathway resulting in increasing cAMP levels. There is a subsequent activation of intracellular cAMP-protein kinase (PK) to an elevation of cAMP-response element-binding protein (CREB) phosphorylation as well as activation of microphthalmia-associated transcription factor (MITF). This latter regulates melanocyte growth, differentiation and function (Figure 1). GPER signaling pathway acts independently from ERs function [76,77]. In vitro GPER agonists inhibit melanoma cell proliferation. [75] GPER co-expressed with ERβ in melanoma have been found to have better outcomes, with lower Breslow thickness, lower mitotic rate and higher presence of peritumoral lymphocyte infiltration [78]

## 5. Present and Future Perspectives

Based on the above evidence, it is possible to postulate that ERβ might have a role in explaining the differences in melanoma prognosis between women and men. As stated previously, studies of estrogen-sensitive cancers showed that loss or decreasing levels of ERβ or an increased ERα to ERβ ratio may be involved in carcinogenesis, thereby suggesting that ERβ has a tumor-suppressive function.

In vitro, the ER antagonist tamoxifen (TAM) has been shown to induce cell death in malignant melanoma cells and to reduce metastatic behavior. Despite these promising results, the treatment of melanoma with TAM has not been shown to be effective in the treatment of advanced melanoma [79]. The effects of chemotherapy with and without TAM for the treatment of aggressive melanoma were compared in different clinical trials. These studies conclude that combination treatment with TAM and chemotherapy may result in improvements in response rates, although it is often accompanied by increased toxicity and no consistent survival benefit [79,80]. A recent meta-analysis, that included nine randomized controlled trials, showed that patients treated with TAM are more likely to respond to chemotherapy. However, no mortality improvement was demonstrated. The authors currently only suggest the use of TAM in clinical trials, given its high hematologic toxicity [80]

It has been hypothesized that tamoxifen decreases cell proliferation when it binds to ERα, while it may increase cell proliferation when it binds to ERβ. Thus, the antitumor vs. pro-survival effects of tamoxifen could depend on the different ERα/ERβ ratios in the given tissue [79,80]. In line with this observation, low levels of expression of ERβ were shown to correlate with tamoxifen resistance in breast cancer cells. There is also epidemiological evidence that women with breast cancer have an increased risk of melanoma and this risk is greater for patients who do not receive anti-estrogen therapy [81,82]. On the contrary, others have found no correlation between receptor beta and Breslow depth or disease stage at diagnosis, and no difference in expression between pregnant and non-pregnant women [83]. TAM likely has more than an ER antagonist action. Indeed, it behaves as an agonist of GPER in vitro and the role of this non-classical estrogen signaling pathway could be crucial to our understanding of its anticancer activity [74].

Endoxifen, an active metabolite of tamoxifen, has been shown to be a safe and effective anti-melanogenic agent in animal models. It exerts its activity by blocking ER transcription. It has been found to be up to 100 times more potent than TAM but with a more favorable toxicity and safety profile. For all these reasons endoxifen could be a promising new therapeutic agent for advanced melanoma [84]. Selective pharmacological activation of ERβ through its agonist LY500307 has been shown to have a potent suppressive action against lung metastasis from melanoma in vitro and in vivo. It acts by recruiting antitumor neutrophils to the metastatic niche though IL-1β, a potent chemotactic factor released from cancer cells upon ERβ stimulation. The activation of ERβ could augment the innate immunity’s ability to suppress cancer metastasis [85]. These pharmacological agents highlight the importance of estrogen signaling in melanoma management and may serve as an important future area of research.

Regarding estrogen signaling and the regulation of the immune system, Natale A. et al. demonstrated through a human-engineered melanoma xenografted into an animal model, that GPER signaling promotes differentiation in melanoma cells, inhibits cancer proliferation, and critically, promotes a cellular phenotype that makes the tumor more susceptible to immune checkpoint blockade [86].

The linkage between melanoma risk and hormonal/reproductive factors requires further investigation. In vitro, sex hormones and gonadotropins stimulate melanogenesis with direct action on the melanocytes. However, in vivo, their antagonists and agonists seem to be effective anticancer drugs. Although these mechanisms alone cannot completely explain all the clinical heterogeneity seen in melanoma cases.

Another issue that requires further investigation is the emerging interplay between microRNAs and estrogen receptor pathways. As is well known, microRNA (miRNA) expression is altered in cancer cells, where its dysregulation can play an oncogenic, metastatic or tumor suppressive role [87]. Several studies have shown the existence of a network involving ERs and miRNAs, either demonstrating a direct effect of estrogen administration in miRNA expression [88] or revealing a direct regulatory effect of miRNAs on ER expression, by directly targeting the 3′-UTR of ESR1/2 mRNAs. In cancer, miRNAs have been observed to be differentially expressed between ER- and ER+ breast cancers [89]. Adams et al. reported the first evidence of the direct regulation of ERα levels by miR-206 in breast cancer [90], an observation that has since been confirmed by other studies. Other research groups have worked with breast cancer cell lines or clinical samples to demonstrate the inhibitory effect caused by miR-22 [91], let-7 [92] or miR-92 [93] overexpression on ERα/β1 levels. This inhibition typically resulted in cell proliferation inhibition and apoptosis activation [92].

Recent studies have demonstrated the cross-talk between miRNAs, long non-coding RNAs (lncRNAs) and coding genes in ER+ and ER− subtypes of breast cancer. The observed networks were closely associated with clinical outcomes and proposed as a prognostic predictor. Specifically, Xiao et al. described that LINC0092 is co-expressed with SFRP1 and RGMA and regulated by miR-449a and miR-452-5p in ER+ breast cancer with better prognosis [94].

ERα-mediated estrogen signaling inhibits the level of miR-21, miR-26a, miR-140, miR-181b and miR-206, and upregulates miR-190a, miR-191, miR-203 and miR-425. Conversely, ERβ is associated with downregulation of miR-17, miR-30a, miR-200a and miR-200b, and overexpression of miR-23b, miR-24-1 and miR-27b [95]. Additionally, specific transcription factors are able to recognize estrogen response elements and activate miRNA transcription in an ER-dependent manner, as described by Nagpal et al. [96].

Estrogen receptors are expressed in different human tissues [97]. Given the tissue specificity of miRNA expression, a complex interplay between miRNAs and ER signaling is likely to exist. In the myometrium, the increased estradiol-17β/ERα in pre-labor inhibits miR-181a, resulting in a further increase in ERα and proinflammatory signals [98]. Conversely, in hepatocellular carcinoma (HCC) miR-18a was identified to target ESR 3′UTR, thus decreasing the ERα protein and stimulating cancer cell proliferation [99]. Additionally, miR-18a and miR-19a, were among the up-regulated miRNAs in MYCN-induced neuroblastoma that targeted and repressed the expression of estrogen receptor-α (ESR1) [100], which is a marker of neuronal differentiation.

A recent study by Vidal-Gómez et al. investigated the pattern of miRNAs induced by estradiol treatment of human umbilical vein endothelial cells (HUVEC) [101]. They described a complex network of up- and down-regulated miRNAs following ERα and ERβ and G protein-coupled estrogen receptor (GPER) stimulation. Given the expression of ERβ observed in melanoma cells, as well as the presence of GPER, it can be postulated that a similar pattern of miRNA activation and repression in the tumor may exist upon estrogen release in the circulation.

Participation in negative and positive feedback loops is another common mechanism of miRNA action in cancer cells, as reported by Di Leva et al. [102]. In this study, miR-221/222 negatively regulate estrogen receptor alpha at the post-transcriptional level, which in turn represses miR-221 and -222 expression. Felicetti et al. indicated that miR-221/222 are key regulators of melanoma development and dissemination [103,104]. In melanoma, miR-221/222 directly modulate KIT oncogene, which is mutated in acral and mucosal melanoma [105]; they also target p27 (CDKN1B) and p57 (CDKN1C) cell cycle inhibitors, thus promoting cell proliferation. We hypothesize that miRNAs play a significant role in co-regulating ERα, KIT, p27 and p57 to increase melanoma cell proliferation.

Recently, Milevskiy et al. described a complex regulatory model of miR-196a locus by ERα. The expression miR-196a is induced by estrogen and was linked to the development of drug-resistance in hormone therapy-responsive breast cancer [106].

The existing scientific literature does not suggest that ERα alone can completely explain melanoma development and progression. Further melanoma-specific studies are required to assess the composite interaction between all types of estrogen receptors, miRNA expression and the impact of both on the tumor microenvironment. The role of miRNA in cancer is an emerging area of research which may provide additional insight into the diagnosis and management of many types of malignancies. Currently, the majority of miRNA research is related to accepted hormone-related malignancies. However, as the evidence of the association between sex hormones and melanoma develops, the role of miRNA may represent a novel area of melanoma research in the future.

## 6. Concluding Remarks

In the era of personalized medicine and diagnostic-therapy, the evaluation of the ER isoforms and GPER expressions in melanoma patients should be considered in order to improve treatment response to novel and innovative therapies. Epidemiological data demonstrate that hormone replacement therapy and the use of the oral contraceptive pill don’t affect the natural history of melanoma and, at present, no association has been found between malignant melanoma risk and use of fertility drugs. Estrogen receptors are important regulators of many molecular pathways and their molecular investigations continue to shed light on our understanding of melanoma genesis and progression in all patients independent of gender.
